# Does clinical decision support system promote expert consensus for appropriate imaging referrals? Chest–abdominal–pelvis CT as a case study

**DOI:** 10.1186/s13244-023-01371-3

**Published:** 2023-03-16

**Authors:** Talya Markus, Mor Saban, Jacob Sosna, Jacob Assaf, Dotan Cohen, Sharona Vaknin, Osnat Luxenburg, Clara Singer, Dorith Shaham

**Affiliations:** 1 Department of Radiology, Hadassah Hebrew University Medical Center, 91120 Jerusalem, Israel; 2 The Gertner Institute for Epidemiology and Health Policy Research, Sheba medical center, Ramat Gan, Israel; 3 Nursing Department, School of Health Professions, Sackler Faculty of Medicine, Tel-Aviv University, Tel-Aviv-Yafo, Israel; 4 Emergency Department, Hadassah Hebrew University Medical Center, Jerusalem, Israel; 5 Medical Technology, Health Information and Research Directorate, Ministry of Health, Jerusalem, Israel

**Keywords:** Appropriateness, Clinical decision support systems, CT scan, ESR iGuide

## Abstract

**Objectives:**

We assessed the appropriateness of chest–abdominal–pelvis (CAP) CT scan use in the Emergency Department (ED), based on expert physicians and the ESR iGuide, a clinical decision support system (CDSS).

**Methods:**

A retrospective cross-study was conducted. We included 100 cases of CAP-CT scans ordered at the ED. Four experts rated the appropriateness of the cases on a 7-point scale, before and after using the decision support tool.

**Results:**

Before using the ESR iGuide the overall mean rating of the experts was 5.2 ± 1.066, and it increased slightly after using the system (5.85 ± 0.911 (*p* < 0.01)). Using a threshold of 5 (on a 7-level scale), the experts considered only 63% of the tests appropriate before using the ESR iGuide. The number increased to 89% after consultation with the system. The degree of overall agreement among the experts was 0.388 before ESR iGuide consultation and 0.572 after consultation. According to the ESR iGuide, for 85% of the cases, CAP CT was not a recommended option (score 0). Abdominal–Pelvis CT was "usually appropriate" for 65 out of the 85 (76%) cases (score 7–9). 9% of the cases did not require CT as first exam modality.

**Conclusions:**

According to both the experts and the ESR iGuide, inappropriate testing was prevalent, in terms of both frequency of the scans and also inappropriately chosen body regions. These findings raise the need for unified workflows that might be achieved using a CDSS. Further studies are needed to investigate the CDSS contribution to the informed decision-making and increased uniformity among different expert physicians when ordering the appropriate test.

## Key points

- Inappropriate testing was prevalent.
- Experts agreement were increased after using the ESR iGuide.
- The use of the ESR iGuide may contribute to informed decision-making.
- The use of the ESR iGuide could increase uniformity among different expert physicians.

## Introduction

CT imaging is a key diagnostic tool, which allows accurate and early diagnosis of many health conditions and pathologies, thus providing better and more appropriate health care. A substantial increase in the use of imaging tests worldwide over the last decades was attributed to the aging population, the increase in the rate of chronic morbidity, the technological advances that enable tests to be performed relatively quickly and simply, and the changes in patients’ perceptions of their own needs [1, 2]. A systematic review published recently found that 20–30% of imaging tests that are conducted neither generate information that improves diagnosis or treatment, nor affect the patient's health—and are therefore unnecessary [3].

Emergency departments (EDs) are sites of prevalent imaging overuse [4]. Over 85% of the emergency physicians believe that too many diagnostic tests are ordered in their own EDs, and 97% stated that at least some of the advanced imaging studies that they personally order are medically unnecessary [5]. Imaging exams referrals in the ED may be considered inappropriate for various reasons, such as not increasing the posttest probability of a diagnosis, being chosen over a more appropriate first exam modality, or not affecting the therapeutic management [6].

Specifically, chest and abdominal complaints are among the principal reasons for ED visits [6]. Imaging studies of the chest, abdomen and pelvis, such as computed tomography (CT), are often ordered to assist in diagnosis and managing potential etiologies.

Lack of awareness of existing guidelines remains a major problem despite the open accessibility of ACR Appropriateness Criteria, developed by the American College of Radiology, and considerable work in introducing them to non-radiology providers [7].

Applying a clinical decision support system (CDSS) can assist clinicians in referring to the appropriate test for the patient’s indication [8].

Most CDSSs perform one of the following operations: First, CDSSs can provide prescribing-based alerts. Second, CDSSs can instigate clinicians to record crucial information in the patient’s electronic health record, in order to meet contractual requirements with the National Health Service. Finally, CDSSs can indicate a patient’s risk of disease either currently (diagnostic) or in a pre-determined timescale (prognostic) [9].

A meta-analysis of Clinical Decision Support Systems (CDSS) concluded that providing context-specific information at the point of care increases 100-fold the odds of providers adopting guideline recommendations [10]. Incorporating a CDSS can assist clinicians and guide them to prescribe the most suitable test and improve patient clinical outcomes alongside optimized resource allocation. The ESR iGuide is an online web portal that recommends the most appropriate imaging tests based on patient data, and it also includes the estimated cost, and expected radiation exposure [11].

Distributed by the ESR's affiliate Quality and Safety in Imaging (QSI) and supported through partnerships with the American College of Radiology and National Decision Support Company, the ESR iGuide was able to rely on extensive experience to develop practical referral guidelines and a tried and tested technical platform. Some recent studies indicated good initial results using this system [12, 13].

For example, Gabelloni et al. [12] assessed the assistance of the ESR iGuide in selecting the most appropriate imaging tests based on clinical signs and symptoms in patients with hepatocellular carcinoma or cholangiocarcinoma. They concluded that the ESR iGuide can be effective in guiding the selection of appropriate imaging tests in these patients and cutting costs due to inappropriate testing. Granata et al. conducted a survey to assess the availability, use, and familiarity of referral guidelines for medical imaging in children and knowledge about the availability of the ESR iGuide among ESR member radiologists. The information gathered confirmed that effective and widespread adoption of imaging referral guidelines is lacking, especially in pediatrics [14].

None of the studies conducted in this era focused on chest–abdominal–pelvis CT scans in the emergency setting in adults. Furthermore, the acceptance rate of CDSS recommendations by clinicians has not been fully elucidated.

Based on a previous study [12, 15], we hypothesized that after using the ESR iGuide system, the agreement level would increase by approximately 20%.

## Aim

To assess the appropriateness rating of Chest–Abdominal–Pelvis Computed Tomography (CT) examinations ordered in the ED, based on expert physicians, before and after using a CDSS tool, namely the ESR iGuide.

## Methods

### Study design

A retrospective cross-study was conducted at a university medical center, based on 100 consecutive medical records of ED patients who underwent non-trauma-based Chest–Abdomen–Pelvis CT scans during December of 2020.

### Data collection

The following information was obtained and presented for each case: patient age, sex, the date and hour of the CT scan referral, the indication of the scan along with relevant clinical information as written on the referral, if contrast was used and how it was administered. In addition, if there had been a prior CT scan, the date of the scan was documented. A summary of the patient’s medical history, ED physical exam findings, and laboratory work and imaging results (other than the CT scan) from their ED stay was also documented from the hospital information system (HIS).

### Procedure

#### Pretest

After receiving ethical approval from our Helsinki committee, four different experts physicians (two radiologists and two emergency medicine physicians with 10–40 years of experience) were asked to rate the appropriateness of the use of a Chest–Abdominal–Pelvis CT for each case, on a scale of 1–7, in which 1 meant “not appropriate” and 7 meant “highly appropriate.” Their decisions were based on the indication as it appeared on the electronic referral and on the electronic medical record, as presented to the investigators by an Excel spreadsheet.

#### ESR iGuide

Simultaneously, we examined the ESR iGuide recommendation for each scenario.

For this purpose, we inserted anonymous case details to the system, including sociodemographic characteristics of the patient (age and gender), clinical indication/s, and also red flags, defined as signs and symptoms found in the patient's history and clinical examination, that help to identify the presence of potentially serious conditions.

We then obtained the recommendations of the ESR-iGuide system, with the correspondent appropriateness rating grade ranging from 9 (highly recommended) to 1 (not recommended). A rating grade of 7–9 corresponded to “usually appropriate”, 4–6 was defined as “may or may not be appropriate”, and a rating of 1–3 was defined as "usually not appropriate". If a CT exam was not part of the recommendations, for the purpose of this analysis, a score of zero was assigned. Seeing as the goal was to explore the most appropriate procedure according to the ESR iGuide, the score for Abdominal–Pelvis CT (without chest), as well as for Chest CT, Abdomen CT and Chest–Abdomen CT were also recorded.

#### Posttest

In a second phase of the investigation performed at least a month later, a screenshot of the ESR iGuide imaging recommendation for each case was added to the spreadsheet that the expert physician had previously received, without inclusion of the previous ratings. The same expert physicians were then asked to perform the same task as before—to rate the appropriateness of each case. This time they knew the ESR iGuide rating and therefore included it in their appropriateness considerations.

### Data analysis

Descriptive measures were calculated for the level of appropriateness of the ED decision for ordering a Chest–Abdominal–Pelvis CT, according to the experts' ratings both prior to ESR iGuide use and after ESR iGuide consultation.

For each clinical case, the mean rate (of four expert physicians) was computed both prior to ESR iGuide use and after ESR iGuide consultation.

For each one of the expert physicians, his/her rating prior to ESR iGuide use was compared to his/her own rating after consultation, using the Wilcoxon signed-rank test. We also used the nonparametric Kolmogorov–Smirnov test when applicable.

The Pearson Correlation coefficient was used to examine the correlation between different pairs of the expert physicians and to explore the association between experts' average rating and ESR iGuide scores.

Agreement between all four expert physicians was also assessed, before and after ESR iGuide consultations. Inter-rater reliability of the rating score was quantified using an intraclass correlation coefficient (ICC) model [16]. Inter-rater reliability of the total rating score was quantified using an ICC model (2,1), namely two-way random effects, absolute agreement and single rates/measurement.

We also explored the degree of agreement between the expert physicians using the Overall Percentage Agreement (OPA). We looked at two different scenarios: a—using the original rating (1–7); b—using a three-class variable (transform 1–2– > 0; 3–5 > 1; 6–7 > 2).

All agreement measures were computed both prior to and after ESR iGuide use. We further performed subanalysis to explore the correlation between expert rate of appropriateness (prior to ESR iGuide consultation) and ESR iGuide appropriateness, and also with other variables: referring physician characteristics [specialty (internal/surgery/emergency), professional status (resident/senior)], patient characteristics (age and gender) and the shift during which the scan was ordered (morning 8:00–16:00, evening 16:00–24:00, night 00:00–8:00).

For subgroup analyses, expert rate of appropriateness was classified using a binary variable (0—non-appropriate, mean rating < 5; 1—appropriate; mean rating ≥ 5). A threshold of 5 was defined by the research group as appropriate using a scale of 1–7, 4 represented the middle point, and 5–7 a "tendency to agree".

Categorical variables were reported as frequency (/percentages) and were compared with Pearson's chi-squared test or Fisher exact test when the value of any expected cell was less than five. For continuous variables such as age, (expressed as mean ± SD) comparisons between the two samples (appropriate/inappropriate) were performed using Independent samples *t* test. A two-tailed *p* value of less than 0.05 was considered significant. Statistical analysis was performed using SPSS V.28 software and SAS enterprise guide v.8.3.

## Results

A total of 100 CT sequential Chest–Abdominal–Pelvic CT examinations performed in the ED were reviewed. The patients’ ages ranged from 20 to 102 years and the mean age was 64.3 ± 19.8 years. Out of 100 patients, 44 were female (44%). The most frequent indications were: cholecystitis/RUQ pain/abnormal liver function tests (LFTs) (21), oncological patients with acute symptoms or clinical deterioration (14), constipation (14), postoperative complications (12), and intestinal obstruction (11). Some form of contrast was used in 80% of the scans (IV 38%, IV + oral 37%, oral 5%), while in 19% of cases no contrast was used (one case did not include information about contrast use).

Regarding the referring physicians who ordered the CT scan, 80 (80%) were residents, while 20% were senior physicians. Of those physicians, 24 (24%) were in the internal medicine discipline, 59 (59%) in surgery, and 17 (17%) in emergency medicine. In terms of the shift during which the CT scan was ordered, 34 (34%) were ordered during the morning shift (8:00–16:00), 47 (47%) during the evening shift (16:00–24:00), and 19 (19%) during the night shift (0:00–8:00).

**Fig. 1:**
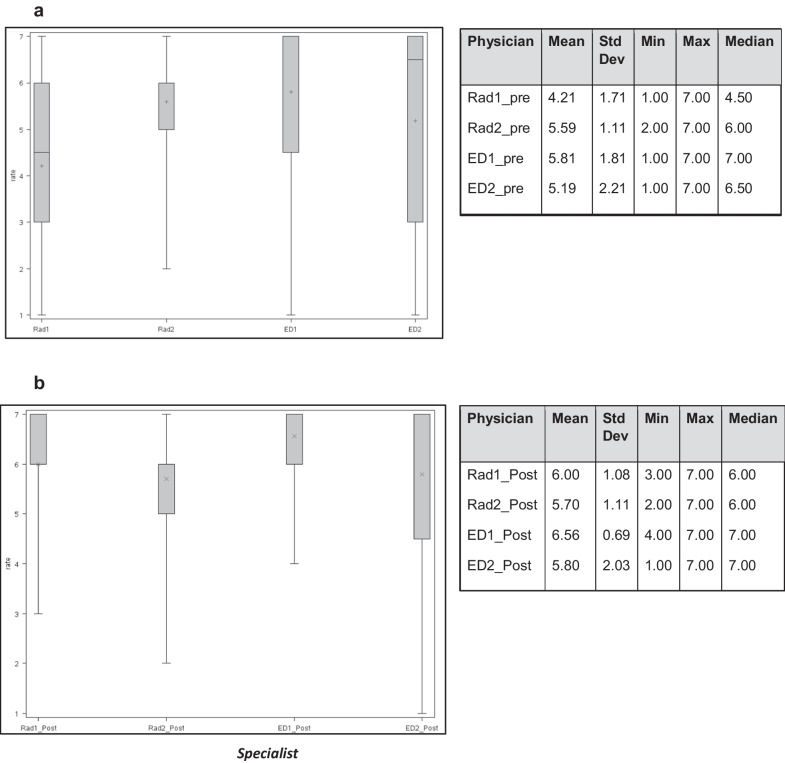
A box plot comparing the ratings of the four physicians prior to ESR iGuide use (**a**) and after ESR iGuide consultation (**b**). *Rad1—Radiologist 1, Rad2—Radiologist 2, ED1—Emergency Department Physician 1, ED2—Emergency Department Physician 2*

Descriptive statistics for the ratings of each expert before and after ESR iGuide use are graphically presented in a box plot in Fig. 1. The rating options (*y* axis) were 1–7, with 1 meaning inappropriate use and 7 meaning appropriate use. As is evident, each expert physician had a distinct pattern to his/her ratings, both before and after using the guidelines.

Prior to ESR iGuide consultation, the median rating varied from 4.5 for one of the radiologists (no.1) to 7 for one of the emergency physicians (no.1), and in general we observe a high diversity in the way that the 4 experts ranked the cases.

The overall mean rating of the four experts’ assessment (average rating per case) was 5.2 ± 1.066, with a median of 5.5, prior to ESR iGuide use. After consulting the ESR iGuide, the overall mean rating of the four experts was 5.85 ± 0.911, with a median of 6.

Based on the mean rate and using a threshold of 5 (a mean rate of 5 and over was considered appropriate, using binary classification), before consulting the ESR iGuide, the experts considered 63% of the tests ordered in the ED as appropriate. After ESR iGuide consultation the percentage of appropriate tests according to the experts increased to 89%.

**Fig. 2:**
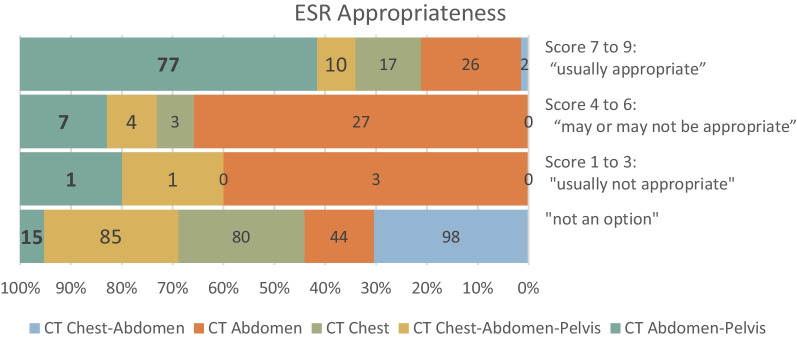
ESR iGuide appropriateness scores for different CT tests (chest–abdomen, abdomen, chest, chest–abdomen–pelvis, abdomen–pelvis)

According to the ESR iGuide system, for 85% of the cases, examination for Chest–Abdominal–Pelvis CT was not an option (score 0), however, the Abdominal–Pelvis protocol was considered "usually appropriate" for 65 out of the 85 (77%) cases (score 7–9). In total, 77% of the cases were found to be "usually appropriate" for CT Abdominal–Pelvis, while for CT abdomen only 26%, and for CT chest only 17%. Nine percent of the cases did not require CT as first imaging modality (Fig. 2).

Each expert physician’s ratings before using the ESR iGuide were then compared to their ratings after consulting the ESR iGuide, using the Wilcoxon Signed-Rank test. This test resulted as significant for 3 out of the 4 expert physicians (except for radiologist #2), indicating that the differences between experts' ratings pre- ESR iGuide consultation and post-ESR iGuide use tend to be different from 0 (Table 1).

**Table 1:** Wilcoxon signed-rank test: pre-post rating

Physician	*S*	Pr ≥|*S*|
Rad1	− 1632	< 0.0001
Rad2	− 144.5	0.3372
ED1	− 381	0.0001
ED2	− 273.5	0.0236

*Rad1* radiologist 1, *Rad2* radiologist 2, *ED1* emergency department physician 1, *ED2* emergency department physician 2

Prior to being shown the ESR iGuide results, the highest Pearson correlation coefficient (when looking at all of the combinations of two of the four expert physicians) was 0.261, indicating negligible correlation between the different pairs of expert physicians. After consulting with the ESR iGuide, the highest Pearson correlation coefficient was 0.407, and five out of the six combinations of pairs had higher correlation after using the ESR iGuide guidelines. Pearson Correlation coefficient between the average expert rating and ESR iGuide scores also increased after ESR iGuide consultation, both for the chest–abdomen–pelvis protocol and the abdomen–pelvis protocol (Table 2).

**Table 2:** Correlation between pairs of physicians’ ratings, and between ESR score (two protocols: abdomen–pelvis, chest–abdomen–pelvis) and average physician rate before and after consulting the ESR iGuide

	Pearson correlation coefficient	
	Before	After
Rad1–Rad2	0.216*	0.377**
Rad1–ED1	0.107	0.054
Rad1–ED2	0.163	0.386**
Rad2–ED1	0.187	0.209*
Rad2–ED2	0.057	0.410**
ED1–ED2	0.261*	0.320*
ESR AP–average 4 experts	0.2*	0.446**
ESR CAP–average 4 experts	0.086	0.197*

*Rad1* radiologist 1, *Rad2* radiologist 2, *ED1* emergency department physician 1, *ED2* emergency department physician 2, *AP* abdomen–pelvis, *CAP* chest–abdomen pelvis

*Statistically significant at the level of *p* = 0.05

**Statistically significant at the level of *p* = 0.0001

The degree of overall agreement (ICC) among the experts was 0.388 before ESR iGuide consultation and 0.572 after consultation. For the original rating of 1–7, the OPA between the four experts was 19.5% before ESR iGuide consultation and 37.67% after consultation. Using a three-class variable (scores 6 and 7 represent agreement), the OPA for the rating between the four experts was 46.5% before ESR iGuide consultation and 70.5% after consultation. Thus, when comparing all four expert physicians at once, we see a small improvement in the correlation and agreement after consultation with the ESR iGuide guidelines. Before consulting with the ESR iGuide, agreement was higher among ED (51%) experts than among radiologists (44%). After consultation the pattern reversed (73% for radiologist vs. 51% for ED) (Table 3).

**Table 3:** Agreement assessment between all four physicians, before and after consulting the ESR iGuide

Statistic	Explanation	PRE	POST
Overall percentage agreement:^a^ ALL	Original rating: 1–7	19.50%	37.67%
Overall percentage agreement:^a^ *Radiologist*	Original rating: 1–7	20%	43%
Overall percentage agreement:^a^ *Emergency Department*	Original rating: 1–7	41%	51%
Overall percentage agreement:^a^ ALL	Three-class variable (1–2; 3–5; 6–7)	46.50%	70.50%
Overall percentage agreement:^a^ *Radiologist*	Three-class variable (1–2; 3–5; 6–7)	44%	73%
Overall percentage agreement:^a^ *Emergency Department*	Three-class variable (1–2; 3–5; 6–7)	51%	51%
Intraclass correlation coefficient (ICC)—absolute	Average measure *(In parenthesis: 95% confidence interval)*	0.388 (0.182–0.555)	0.572 (0.418–0.694)

^a^See “Appendix” for calculations

No significant association was found between the degree of expert physician appropriateness assessment and the gender of the patient, the shift during which the scan was ordered or the referring physician’s status. A significant association (*p* = 0.04) was found between the specialty of the physician who requested the scan classified as surgery/non-surgery and degree of expert physician assessment, where proportion of appropriate CT scan is 71% for surgery versus 51% for non-surgery. There was no statistically significant difference in the mean age between patients within experts' appropriate/ non-appropriate categories (mean age 65.3 ± 18.5 years vs. 61.9 ± 22 years, respectively) (Table 4).

**Table 4:** Analysis of CT orders regarding Experts’ Assessment of Appropriateness (prior to ESR): Pearson chi-square test /Fisher's exact test

Variable	Appropriate (*n* = 63)	Non-appropriate (*n* = 37)	*p* value
*Shift during which the scan was ordered*			0.42
Morning	24/34 (70.6%)	10/34 (29.4%)	
Evening	29/47 (61.7%)	18/47 (38.3%)	
Night	10/19 (52.6%)	9/19 (47.4%)	
*Specialty of the physician who requested the scan*			0.04*
Surgery	42/59 (71.2%)	17/59 (28.8%)	
Non-surgery	21/41 (51.2%)	20/41 (48.8%)	
*Physician’s status*			0.75
Resident	51/80 (63.7%)	29/80 (36.2%)	
Senior	12/20 (60%)	8/20 (40%)	
*Gender*			0.17
Female	31/44 (70.4%)	13/44 (29.5%)	
Male	32/56 (57.1%)	24/56 (42.9%)	
^**a**^Age	18.5 ± 65.3	22 ± 61.9	0.41

^**a**^Two-sided *t* test

*Statistically significant at the level of *p* = 0.05

Explanations for the disagreement were provided by two out of the four experts. The most common comments that we received were regarding body region imaging (i.e., a referral for CAP CT rather than abdominal or abdominal–pelvis scan). For example, in case 27, the radiology expert pointed out a grading error, that a Chest CT scan should have been performed, and not CAP as referred.

## Discussion

The current study aimed to assess the appropriateness of the use of Chest–Abdominal–Pelvis CT scans in the ED, before and after using a CDSS tool, namely the ESR iGuide. The main findings illustrated that before using the ESR iGuide, based on a threshold of 5, the experts considered 63% of the tests as appropriate. This number increased by 25% after consultation with the system (89%). According to the ESR iGuide, in 85% of the cases the full Chest–Abdominal–Pelvis protocol (three different regions) was considered inappropriate. In 65 out of those 85 cases, the system recommended performing only an Abdominal–Pelvic exam.

CDSS are used to augment clinicians in their complex decision-making processes and intend to improve healthcare delivery by enhancing medical decisions with targeted clinical knowledge, patient information, and other health information. As the prevalence of unnecessary imaging use is increasing, developing an efficient form of such technology is crucial.

A recent study found that the prevalence of inappropriate examinations in the ED was 36.3% for CT and 84.4% for the US for patients admitted with an abdominal complaint [17], based on evidence-based recommendations by the American College of Radiology. Similar rates were found in this research for the agreement of the experts; the prevalence of inappropriate decisions in the ED was 37%.

Another study explored the frequency of unindicated CT phases in women of reproductive age who have undergone CT for non-traumatic abdomino-pelvic emergencies. Out of 197 cases, 93 (47%) were unindicated with an average of 1.2 inappropriate phases per patient [18].

The overall mean rating of the four experts (average rating per case) was medium–high, but with a low score for inter-rater agreement that increased after ESR iGuide consultation. These results are in-line with a previous study that aimed to assess the acceptance and reliability of the ESR iGuide. The study included four experts who were asked to rate 40 simulated clinical cases on a 5-level scale, for the level of agreement with the ESR iGuide’s recommended procedures. Study findings showed that all expert physicians totally agreed with the system recommendation in 75% of cases with ICC of 0.35 [19].

Proportion of agreement with ED decision to perform Chest–Abdominal–Pelvis CT scan was higher for referrals by surgeons than by non-surgeons, including internal medicine and emergency medicine. This was expected as surgery clinical cases are usually more straightforward (ex. Query for Appendicitis) while internal medicine deals with "less well-defined problems" where CT is not always a first choice.

In-line with our results, Young et al. [20] found that primary care physicians (internal medicine and family medicine) were almost twice as likely to order an inappropriate MRI as orthopedists, neurologists and surgeons.

Along with a personal responsibility for patients, primary care physicians see a wide range of clinical conditions for the patients they treat and play a gatekeeper role that involves assessing which patients should be referred for imaging and specialist health services. This role can be difficult for primary care physicians to maintain, and fear of conflict can make it difficult for them to resist requests from patients for referrals that are not medically justified [21].

Based on expert physicians, for 15 cases out of 100, a low appropriateness assessment (mean physicians' rate < 4) was found. Most of them were exams for patients with unspecified abdominal pain, suspected renal failure or fatigue/malaise. On the other hand, the highest appropriateness assessment (mean physicians' rate > 6.5) was found for patients with symptoms suggestive of appendicitis, peritonitis, intestinal obstruction and cirrhosis.

These results are inconsistent with previous findings. Francisco et al. [17] found that the most frequent inappropriate uses of CT were evaluation of biliary disease, pancreatitis, renal failure, and uncomplicated pyelonephritis. Other less common inappropriate orders were related to the lack of intravenous (IV) contrast when it is usually indicated (acute abdominal pain, small bowel obstruction, diverticulitis, appendicitis), or using IV contrast when it is not indicated (e.g., urolithiasis). However, Giannitto et al. [18] found that unindicated scans were most frequent for appendicitis and unlocalized abdominal pain.

When examining the justification of the exams performed in the ED compared to the ESR iGuide recommendations, the results are less encouraging. According to ESR iGuide appropriateness criteria, in a high proportion of cases the addition of chest to the abdominal–pelvis protocol was unnecessary, thus increasing unnecessarily the area with radiation. Thus, another category of importance in future analysis is not only unnecessary studies but rather inadequate coverage of the scanned areas.

There are several possible explanations for this observation. First, this protocol is commonly used in the ED. Therefore, referring physicians may be less diligent requesting only abdominal–pelvis CT compared to chest–abdominal–pelvis CT, which would yield a higher rate of inappropriate orders for the latter [2].

In addition, due to high availability of CT scans in the ED, it becomes an immediate tool compared to other imaging means.

Another explanation would be the heterogeneous profile of clinical indications that lead referral physician to order this imaging modality [22].

Subanalysis to explore the correlation between ESR iGuide appropriateness for Chest–Abdominal–Pelvis CT scan and referral physician characteristics (resident/senior), patient characteristics and the shift during which the scan was ordered resulted in no significant results. This was expected, since chest–abdominal–pelvis CT was found inappropriate for 85 of the cases. Given that most radiation exposure is now from man-made sources and that most of this exposure originates from medical imaging, it is important to recognize the possible effects and risks of this radiation exposure to the human body and utilize available systems, such as CDSS, to reduce it.

Reducing the number of unnecessary tests is also important in the radiologist-shortage era. As the number of imaging studies increases by a rate of up to five percent per year, the numbers of radiology residency positions only increases by two percent [23]. This shortage has a dramatic impact on patient care, especially in the ED. A shortage of radiologists leads to increased turnaround times for study results to be generated, which can negatively impact patient outcomes.

## Study limitations

Our study has several limitations. The expert physicians who performed the rating were exposed only to the indication as it appears on the referral and on the medical chart, without facing the patients themselves. Moreover, expert physicians were asked to rank the appropriateness of ED decision of performing CT for Chest–Abdominal–Pelvis from 1 to 7, 1 meaning usually not appropriate and 7 meaning very appropriate, but no exact guidelines to the meaning of each score were provided, and specifically what score to give if CT for Chest–Abdominal–Pelvis was not appropriate, but an Abdominal–Pelvic CT was indeed appropriate. This may be a source for disagreement between different expert physicians.

Third, due to lack of documentation, we could not take into account that communication between radiologists and clinicians is often based not only on written referrals, but also on direct or telephone contact.

Finally, the study findings are limited by the small number of cases and by a specific scenario being analyzed.

## Conclusions

A low level of agreement was observed between the different expert rates. Based on mean rating, before ESR iGuide consultation the prevalence of inappropriate decisions at the ED was 37% according to expert physicians' opinions. According to the ESR iGuide score recommendation, 85% of the ordered tests were not appropriate, of which 65 did not require the inclusion of the chest part in the protocol. Inter-rater agreement between expert physicians increased after ESR iGuide consultation. We believe that use of the ESR iGuide will contribute to informed decision-making and increased uniformity among different expert physicians and that more appropriate testing would decrease unnecessary radiation in general and specifically to unindicated body parts.

## Data Availability

The authors confirm that the data supporting the findings of this study are available within the article.

## Appendix

See Table 5.

**Table 5:** Overall percentage agreement (ID expert: 1,2- radiology, 3,4- Emergency Department Physician) before and after consulting the ESR iGuide

Measure	Difference pair 1 and 2	Difference pair 1 and 3	Difference pair 2 and 3	Difference pair 1 and 4	Difference pair 2 and 4	Difference pair 3 and 4
*A. Before consulting the ESR iGuide*						
*Original rating (Range 1–7)*						
Total count of 0 in difference column	20	10	13	13	20	41
Total ratings	100	100	100	100	100	100
Proportion agreement	0.2	0.1	0.13	0.13	0.2	0.41
Percentage agreement	20%	10%	13%	13%	20%	41%
Overall percentage agreement	19.50%					
Total count of 0 in difference column	44	36	57	37	54	51
Total ratings	100	100	100	100	100	100
Proportion agreement	0.44	0.36	0.57	0.37	0.54	0.51
Percentage agreement	44%	36%	57%	37%	54%	51%
Overall percentage agreement	46.50%					

Measure	Difference pair 1 and 2	Difference pair 1 and 3	Difference pair 2 and 3	Difference pair 1 and 4	Difference pair 2 and 4	Difference pair 3 and 4
*B After consulting the ESR iGuide*						
*Original rating (Range 1–7)*						
Total count of 0 in difference column	43	42	36	33	21	51
Total ratings	100	100	100	100	100	100
Proportion agreement	0.43	0.42	0.36	0.33	0.21	0.51
Percentage agreement	43%	42%	36%	33%	21%	51%
Overall percentage agreement	37.67%					
Total count of 0 in difference column	73	78	70	68	63	71
Total ratings	100	100	100	100	100	100
Proportion agreement	0.73	0.78	0.7	0.68	0.63	0.71
Percentage agreement	73%	78%	70%	68%	63%	71%
Overall percentage agreement	70.50%					

Calculations shown for original rating and for three-class variable

Three-class variable (Range 0–2; Transform 1–2- > 0; 3- 5 > 1; 6–7 > 2)

## Abbreviations

ACR: American College of Radiology
CDSS: Clinical decision support systems
CT: Computed tomography
ICC: Intraclass correlation
